# A stabilized spatiotemporal kriging method for disease mapping and application to male oral cancer and female breast cancer in Taiwan

**DOI:** 10.1186/s12874-022-01749-9

**Published:** 2022-10-13

**Authors:** Dai-Rong Tsai, Jing-Rong Jhuang, Shih-Yung Su, Chun-Ju Chiang, Ya-Wen Yang, Wen-Chung Lee

**Affiliations:** 1 Institute of Epidemiology and Preventive Medicine, College of Public Health, National Taiwan University, Rm. 536, No. 17, Xuzhou Rd., Taipei, 100 Taiwan; 2 Taiwan Cancer Registry, Taipei, Taiwan

**Keywords:** Stabilized spatiotemporal kriging, Disease map, Incidence, Oral cancer, Breast cancer

## Abstract

Mapping spacetime disease rates can provide a more in-depth understanding of their distribution and trends. Traditional spatiotemporal kriging methods can break the constraints of geopolitical boundaries and time intervals. Still, disease rates in densely and sparsely populated areas are stabilized to the same degree, resulting in a map that is oversmoothed in some places but undersmoothed in others. The stabilized spatiotemporal kriging method proposed in this study overcomes this problem by allowing for nonconstant variances over space and time. A spatiotemporal map of the standardized incidence ratio for oral cancer in men in Taiwan between 1997 and 2017 reveals that the high-risk areas for oral cancer are in the midwestern and southeastern regions of Taiwan, spreading toward the center and north, with persistent cold spots in the northern and southwestern urban regions. However, the corresponding map for breast cancer in women in Taiwan reveals that the high-risk areas for breast cancer are concentrated in densely populated urban regions in the west. Spatiotemporal maps facilitate our understanding of disease risk dynamics. We recommend using the proposed stabilized spatiotemporal kriging method for mapping disease rates across space and time.

**Supplementary Information:**

The online version contains supplementary material available at 10.1186/s12874-022-01749-9.

## Introduction

Disease mapping has long been a part of public health. Epidemiologists often construct disease maps to investigate the geographical variation in diseases. The choropleth map is the widely used map type for mapping disease rates, using local administrative areas (LAAs) such as counties, cities, or towns as geographic units. The isarithmic map was another common map type, using latitude and longitude as geographic units and breaking the constraints of geopolitical boundaries. Tracking the disease rates of each geographic unit over several years constitutes spatiotemporal data. The three-dimensional spatiotemporal map visualizes the distribution of disease risks over space and time, which can help detect possible risk factors and form the basis of health policies.

Mapping disease rates with raw data and visualizing with the choropleth map may incur too much spatial variability; in LAAs with smaller populations, the disease rates may become highly unstable. Empirical Bayes methods stabilize the rates in LAAs adaptively according to their population size [1]. Conditional autoregressive models further consider spatial correlations between LAAs [2]. The spatial random effects model involves the benefits of the abovementioned methods and can deal with confounding factors [3, 4]. These methods can also incorporate “time” as a covariate or as a random effect to deal with the variations in disease risks in the time domain [3, 4].

Mapping disease rates as a continuous surface and visualizing with the isarithmic map can more legibly depict the spatiotemporal patterns of disease risks. Trend surface analysis and kernel smoothing created a continuous surface of disease rates [5–7]. The spatiotemporal kriging method has been widely used in environmental and earth sciences [8–10]. Epidemiologists also applied the kriging method to investigate the spread of infectious diseases [11–13], the distribution of environmental risk factors [14, 15], and the spatiotemporal variations of disease rates [16, 17]. The kriging method estimates the spatiotemporal correlations based on a nonparametric model and extrapolates the disease rates at any given point in space and time. The method breaks the constraints of geopolitical boundaries and time intervals [18–20]. However, because of the homoscedasticity assumption, the kriging method stabilizes the disease rates in LAAs with different population sizes to the same degree, resulting in a map that is oversmoothed in some places but undersmoothed in others.

This study proposed a stabilized spatiotemporal kriging method for disease mapping. This method accounts for the heteroscedasticity in the spatiotemporal data, permitting the disease rates to be shrunk toward the mean to different degrees, thus avoiding oversmoothing and undersmoothing. Monte Carlo simulations were conducted to assess the properties of the proposed method compared with the traditional kriging method. Real data applications for oral cancer in men and breast cancer in women in Taiwan were also performed for demonstration.

## Methods

### Stabilized spatiotemporal kriging

Let the observed and expected number of cases for a particular disease in the $i$ th LAA of the $j$ th year be ${O}_{ij}$ and ${E}_{ij}$, respectively ($i=1,\dots ,N$; $j=1,\dots ,M$). The expected number of cases can be calculated as

$$E_{ij}={\textstyle\sum_{k=1}^K}p_{ijk}\times R_k,$$

where ${p}_{ijk}$ is the population size in the $i$ th LAA of the $j$ th year for the $k$ th age group and ${R}_{k}$ is the incidence rate for the $k$ th age group of a standard population ($k=1,\dots ,K$). The standardized incidence ratio, SIR, and its natural logarithm, logSIR, are then estimated as ${\widehat{r}}_{ij}=\frac{{O}_{ij}}{{E}_{ij}}$ and ${\widehat{\theta }}_{ij}={log}_{e}\left({\widehat{r}}_{ij}\right)$, respectively. We assume a linear mixed model as follows:

1 $${\widehat{\theta }}_{ij}={\theta }_{ij}+{\varepsilon }_{ij}=\mu +{\delta }_{ij}+{\varepsilon }_{ij},$$

where ${\theta }_{ij}$ is the natural logarithm of the true standardized incidence ratio (logSIR) and ${\varepsilon }_{ij}$ is the measurement error, which is unbiased and independent of one another; thus, $E\left({\varepsilon }_{ij}\right)=0$ ($i=1,\dots ,n$; $j=1,\dots ,m$) and $Cov\left({\varepsilon }_{ij},{\varepsilon }_{kl}\right)=0$ ($ij\ne kl$). The ${\theta }_{ij}$ is the sum of the spatiotemporal mean ($\mu$, a fixed effect) and the spatiotemporal variations (${\delta }_{ij}$, random effects with a mean of zero). The ${\delta }_{ij}$ is second-order stationary and spatially isotropic; accordingly, the variance of ${\theta }_{ij}$ is a constant ${\tau }^{2}$ and the covariance between ${\theta }_{ij}$ and ${\theta }_{kl}$ (${C}_{ij,kl}$, $ij\ne kl$) depends only on the spatial distance (${h}_{ik}$; unit: km) between the centroids of the $i$ th and $k$ th LAAs and the temporal distance (${u}_{jl}$; units: year) between the $j$ th and $l$ th years. Commonly used distance metric in health research involved straight line (Euclidean) distance and Manhattan distance, and, in this study, we used the Euclidean distance.

Data consists of $n\times m$ couples of $\left({\widehat{\theta }}_{ij},{\widehat{v}}_{ij}\right)$ for $i=1,\dots ,n$ and $j=1,\dots ,m$. The ${\widehat{v}}_{ij}$, the estimation of $Var\left({\varepsilon }_{ij}\right)$, is $\frac{1}{{O}_{ij}}$ (Additional file 1: Appendix 1). Under the formula (1), a best linear unbiased predictor for the logSIR in a particular locality (subscript $s$; this can be an LAA centroid or any noncentroid geographical coordinate) at one specific time point (subscript $t$; this can be the middle or any time point in any year) is.

$${\widehat\theta}_{st}={\textstyle\sum_{i=1}^n}{\textstyle\sum_{j=1}^m}w_{ij}{\widehat\theta}_{ij},$$

and the weights ${w}_{ij}$ are

2 $$\left[\begin{array}{c}{w}_{11}\\ {w}_{12}\\ \vdots \\ {w}_{nm}\\ \lambda \end{array}\right]={\left[\begin{array}{ccccc}{\tau }^{2}+{\widehat{v}}_{11}& {C}_{\mathrm{11,12}}& \cdots & {C}_{11,nm}& 1\\ {C}_{\mathrm{12,11}}& {\tau }^{2}+{\widehat{v}}_{12}& \cdots & {C}_{12,nm}& 1\\ \vdots & \vdots & \ddots & \vdots & \vdots \\ {C}_{nm,11}& {C}_{nm,12}& \cdots & {\tau }^{2}+{\widehat{v}}_{nm}& 1\\ 1& 1& \cdots & 1& 0\end{array}\right]}^{-1}\left[\begin{array}{c}{C}_{11,st}\\ {C}_{12,st}\\ \vdots \\ {C}_{nm,st}\\ 1\end{array}\right],$$

where $\lambda$ is the Lagrange multiplier to ensure $\sum_{i=1}^{n}\sum_{j=1}^{m}{w}_{ij}=1$.

The parameters ${\tau }^{2}$ and ${C}_{ij,kl}$ (${C}_{ij,kl}={C}_{kl,ij}$; $i,k=1,\dots ,n$; $j,l=1,\dots ,m$; $ij\ne kl$) in formula (2) can be estimated from a semivariogram. First, we calculate a total of $\frac{1}{2}\times n\times m\times \left(n\times m-1\right)$ paired sample semivariances: $\frac{1}{2}{\left({\widehat{\theta }}_{ij}-{\widehat{\theta }}_{kl}\right)}^{2}$ for $i,k=1,\dots ,n$; $j,l=1,\dots ,m$; $ij\ne kl$. We then produce the semivariogram by plotting these semivariances against the spatial distance ${h}_{ik}$ and temporal distance ${u}_{jl}$ between any pair of distinct spatiotemporal data points. We then fit a spatiotemporal semivariance function $\gamma \left(h,u\right)$, which monotonically increases as $h$ and $u$ increase, to the semivariogram based on generalized least squares. Herein, we use the “sum-metric” spatiotemporal semivariance structure detailed in [21]: $\gamma \left(h,u\right)={\gamma }_{S}\left(h\right)+{\gamma }_{T}\left(u\right)+{\gamma }_{ST}\left[\sqrt{{h}^{2}+{(\alpha \times u)}^{2}}\right]$, where $\gamma_S\left(\cdot\right)$*,* $\gamma_T\left(\cdot\right)$, and $\gamma_{ST}\left(\cdot\right)$ are the semivariance functions for space, time, and spacetime interactions, respectively, and $\alpha$ is a parameter that integrates the distances in space and time into a single spacetime distance metric. Note that the functions $\gamma_S\left(\cdot\right)$, $\gamma_T\left(\cdot\right)$, and $\gamma_{ST}\left(\cdot\right)$ must increase monotonically and have upper limits so that the spatiotemporal semivariance function $\gamma \left(h,u\right)$ can approach a maximum (referred to as the “sill”) when the distances in space and time become extremely large. Commonly used semivariance functions include the spherical, exponential, and Gaussian functions.

We derive the covariance function $C\left(h,u\right)$ by subtracting the semivariance function from the sill. This function is then a monotonically decreasing function of distances in both space and time. The parameters in formula (2) are then estimated as ${\widehat{\tau }}^{2}=C\left(0, 0\right)$, ${\widehat{C}}_{ij,kl}=C\left({h}_{ik},{u}_{jl}\right)$, and ${\widehat{C}}_{ij,st}=C\left({h}_{is},{u}_{jt}\right)$, respectively, for $i,k=1,\dots ,n$; $j,l=1,\dots ,m$; $ij\ne kl$.

We refer to this method as stabilized spatiotemporal kriging. The key to stabilization is that with the error variances, ${v}_{11},\dots ,{v}_{nm}$, added to the diagonal of the square matrix in formula (2), smaller weights are given to data with larger variances and larger weights to those with smaller variances. The approach extends the stabilized kriging method in a purely spatial setting, as proposed by Hsu et al. [22], to deal with spacetime data.

The stabilized spatiotemporal kriging method can be implemented through the following steps:

1. Produce a semivariogram and fit the semivariance function. This can be achieved using the same software package used for traditional spatiotemporal kriging.
2. Subtract the semivariance function from the sill (the maximum of the semivariance function) and derive the covariance function.
3. Estimate the parameters ${\tau }^{2}$, ${C}_{ij,kl}$, and ${C}_{ij,st}$ (${C}_{ij,kl}={C}_{kl,ij}$; $i,k=1,\dots ,n$; $j,l=1,\dots ,m$; $ij\ne kl$) from a direct readout of the values from the covariance function.
4. Calculate the kriging weight for any spatiotemporal point to be estimated. This can be done using any statistical package that supports formula (2) matrix calculations.
5. Calculate the weighted average and then use exponentiation to obtain the kriging estimate of SIR at that spatiotemporal point.

We further use the annual percent change (APC) to measure the trend of SIR over time. We recommend using Cole’s definition of the symmetric percentage change [23]. The APC at a certain spatiotemporal point, $st,$ can be obtained as follows:

1. Calculate the kriging estimates of logSIR at the spatial point $s$ within a 5-year time window centered at the temporal point $t$.
2. Perform a simple linear regression of the kriged logSIR against time (in years) to obtain the estimate of the slope $\widehat{\beta }$.
3. The APC at the spatiotemporal point $st$ is $\widehat{\beta }\times 100 (\mathrm{\%})$.

A positive APC indicates that the value of SIR is increasing, a negative APC that the value is declining, and an APC near zero that the value remains stable. Note that the conventional definition of APC leads to overt asymmetry; the value comparing two consecutive years is $\left[\mathrm{exp}\left(\widehat{\beta }\right)-1\right]\times 100\mathrm{\%}$ (with a lower limit of $-100\mathrm{\%}$ and without an upper limit) with the first year as the reference, but is $\left[1-\mathrm{exp}\left(-\widehat{\beta }\right)\right]\times 100\mathrm{\%}$ (with an upper limit of $100\mathrm{\%}$ and without a lower limit) with the second year as the reference. However, Cole’s definition of APC is always $\widehat{\beta }\times 100\mathrm{\%}$.

### Monte Carlo simulation

We generated $4417\times 21$ spatiotemporal points (with longitudes between 120.00° and 121.95° and latitudes between 21.92° and 25.27° from 2000 to 2020) to encompass the entire Taiwan island (Additional file 1: Appendix 2). We considered a single-hotspot scenario at coordinate point ℎ and a double-hotspots scenario at coordinate points ℎ and ℎ^*^, respectively (Additional file 1: Appendix 3). We set the true value of the logSIR for each spatiotemporal point according to the two-dimensional Gaussian function, which diminishes in value with distance and time, as follows:

1. A single hotspot at coordinate point ℎ

$${\theta }_{st}=\mathrm{log}\left(1.0\right)+\mathrm{exp}\left(-\frac{1}{2}{\left(\frac{{D}_{s,h}}{50-9t}\right)}^{2}\right),$$

2. Double hotspots at coordinate points ℎ and ℎ^*^, respectively,

$${\theta }_{st}=\mathrm{log}\left(1.0\right)+\mathrm{exp}\left(-\frac{1}{2}{\left(\frac{{D}_{s,h}}{50-9t}\right)}^{2}\right)+\mathrm{exp}\left(-\frac{1}{2}{\left(\frac{{D}_{s,{h}^{*}}}{50-9t}\right)}^{2}\right),$$

where ${D}_{a,b}$ denotes the distance between two coordinate points *a* and *b* (unit: km).

We used a log-normal distribution to generate the population size of the 349 LAAs in Taiwan. We considered the expected values of the population size were 50,000 and 25,000; the coefficients of variation of the population size were 0.5 and 1.0. For an LAA, we multiplied its simulated population size by the simulated disease rate (assumed to follow an exponential distribution) at the centroid of that LAA to obtain the expected number of cases (${E}_{ij}$). We then used a Poisson distribution (with a mean of ${E}_{ij}\times {e}^{{\theta }_{ij}}$) to generate the observed number of cases (${O}_{ij}$) for each LAA. The estimated logSIR (${\widehat{\theta }}_{ij}$) of the LAA is the logarithm of the ratio between the observed and expected numbers of cases.

We used the stabilized spatiotemporal kriging method and the traditional spatiotemporal kriging method to estimate the logSIR of all coordinate points within the geopolitical boundary of Taiwan based on the simulated data. We calculated the index of symmetric mean absolute percentage error (SMAPE):

$$\mathrm{SMAPE}=\frac{100\%}{21\times4417}{\textstyle\sum_{t=1}^{21}}{\textstyle\sum_{s=1}^{4417}}\frac{\vert e^{{\widehat\theta}_{st}}-e^{\theta_{st}}\vert}{e^{{\widehat\theta}_{st}}+e^{\theta_{st}}}.$$

The smaller the SMAPE index, the closer the estimated value is to the true value. We performed 1,000 simulations for each scenario.

All analyses were performed using SAS version 9.4 and R version 3.5.2. R packages, including gstat, sp, and spacetime, were used to perform the spatiotemporal kriging method.

## Results

### Simulation results

The simulation results are presented in Table 1. The SMAPEs increased with decreasing population size and increasing coefficient of variation for both methods. For the single-hotspot scenario, the stabilized spatiotemporal kriging method and the traditional kriging method had similar performance. For the double-hotspots scenario, the stabilized spatiotemporal kriging method performed better (having smaller SMAPE) than the traditional spatiotemporal kriging method in all combinations of population size and coefficient of variation.

**Table 1:** The symmetric mean absolute percentage errors (%) of the traditional and stabilized spatiotemporal kriging in the scenarios of a single hotspot and double hotspots

		A single hotspot		Double hotspots	
Population size	Coefficient of variation	Traditional spatiotemporal kriging	Stabilized spatiotemporal kriging	Traditional spatiotemporal kriging	Stabilized spatiotemporal kriging
25,000	0.5	21.18	21.02	26.11	20.75
25,000	1.0	21.56	21.36	33.31	28.87
50,000	0.5	15.26	15.12	24.53	20.69
50,000	1.0	18.23	18.08	24.99	20.14

### Real data applications

We used oral cancer in men and breast cancer in women in Taiwan as examples to demonstrate the methodology. The data of male patients with oral cancer and female patients with breast cancer from 1997 to 2017, including their ages and the LAAs they resided in, were extracted from the Taiwan Cancer Registry [24, 25]. The age-specific mid-year population numbers (the averages of the end-year population numbers in two consecutive years) for men and women in every LAA in Taiwan from 1997 to 2017 were extracted from an online database provided by the Department of Household Registration in Taiwan’s Ministry of the Interior. We focused our analysis on the 349 LAAs on the main island of Taiwan (the 19 LAAs in the outlying islands were excluded). The patients’ ages were divided into 18 age groups: 0–4, 5–9, …, 80–84, and 85 + . The average incidence rate for each age group on the main island over 21 years (1997–2017) was taken as the age-specific rate of the standard population. A total of $2\times 349\times 21=\mathrm{14,658}$ SIRs (2 cancer types, 349 LAAs, 21 years) were then calculated as described previously. In this study, the representative point of an LAA was taken to be its geometric center and the middle of each year, July 1.

**Fig. 1:**
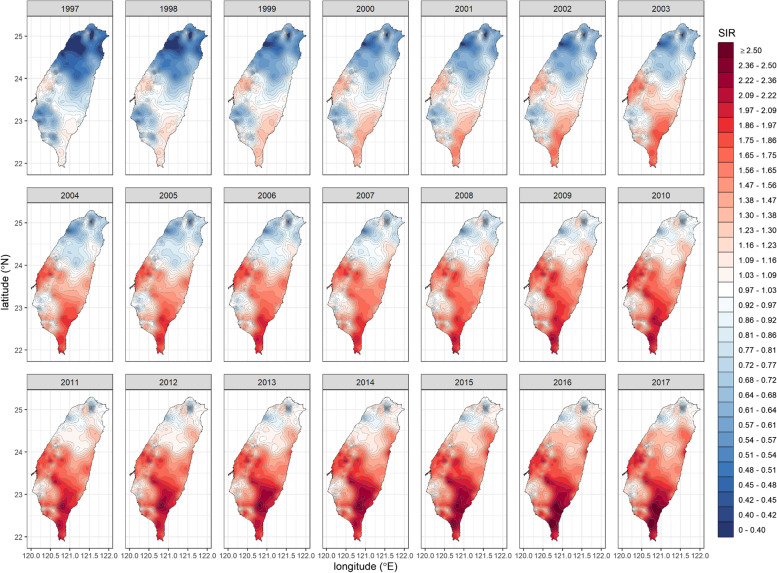
The stabilized spatiotemporal kriging map for the standardized incidence ratios (SIRs) of oral cancer in men in Taiwan, 1997 to 2017

**Fig. 2:**
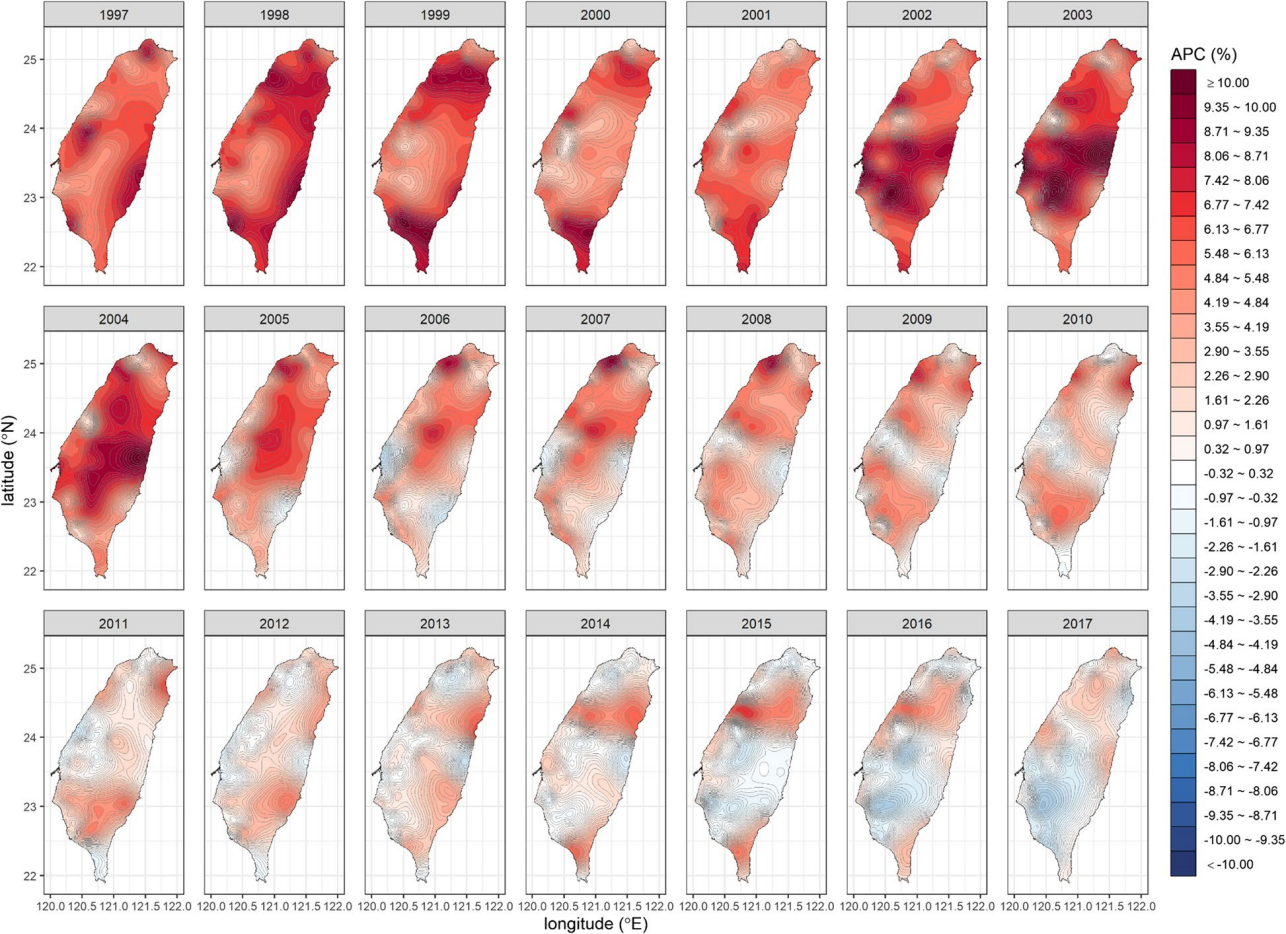
The stabilized spatiotemporal kriging map for the annual percent changes (APCs) in the standardized incidence ratios of oral cancer in men in Taiwan, 1997 to 2017

**Fig. 3:**
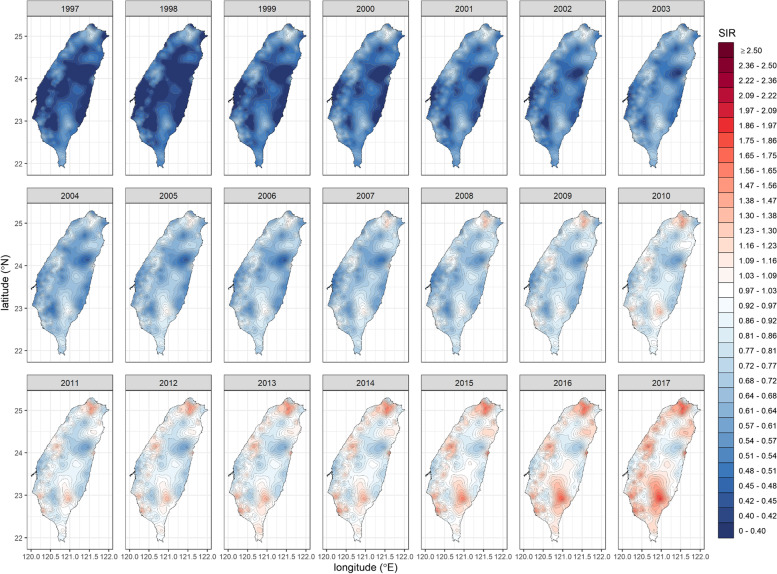
The stabilized spatiotemporal kriging map for the standardized incidence ratios (SIRs) of breast cancer in women in Taiwan, 1997 to 2017

**Fig. 4:**
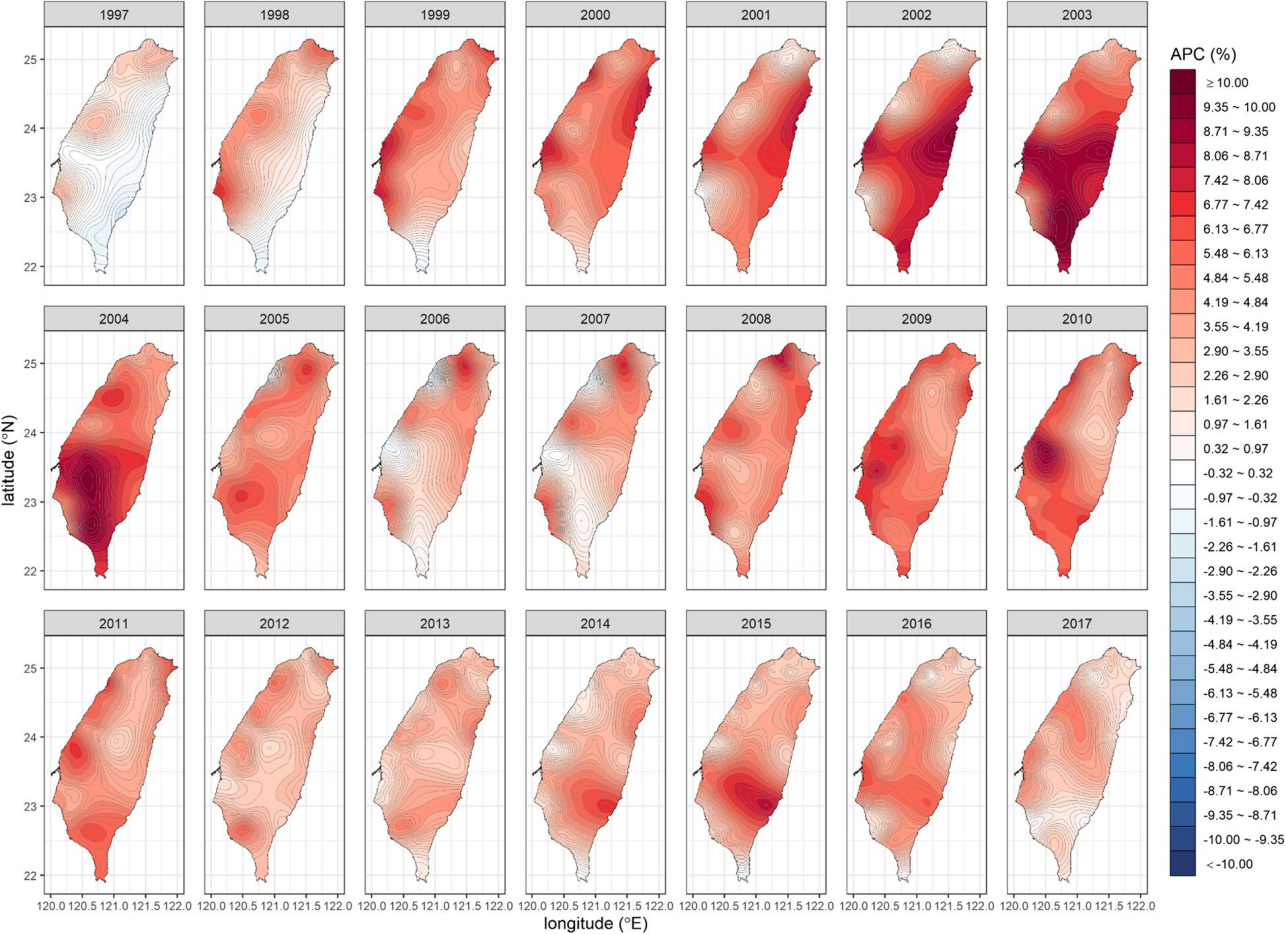
The stabilized spatiotemporal kriging map for the annual percent changes (APCs) in the standardized incidence ratios of breast cancer in women in Taiwan, 1997 to 2017

We generated a square mesh coordinate point system with a 1-km spacing (69,084 coordinate points, with longitudes between 120.00° and 122.02° and latitudes between 21.89° and 25.31°) to encompass the entire main island of Taiwan. We then used the proposed stabilized spatiotemporal kriging method to estimate the SIRs of all the coordinate points on the main island (a total of 32,263 coordinate points) for a total of $21\times 12=252$ months (each month represented by its first day) based on the available data of $349\times 21=\mathrm{7,329}$ couples of $\left({\widehat{\theta }}_{ij},{\widehat{v}}_{ij}\right)$ for $i=1,\dots ,349$ and $j=1,\dots ,21$. We applied the sum-metric structure and used the spherical spatiotemporal semivariance function. We also calculated the APC at each spatiotemporal point using kriged SIRs. Finally, we produced the spatiotemporal SIR and APC maps for oral cancer in men (Figs. 1 and 2; Additional file 1: Appendix 4) and breast cancer in women in Taiwan (Figs. 3 and 4; Additional file 1: Appendix 5).

The SIR map for oral cancer in men (Fig. 1) reveals that before 2000, people in most parts of Taiwan, except for the midwestern and southeastern regions, were at low risk of oral cancer, with cold spots in the north and southwest. After 2000, high-risk areas began to emerge in the Central and Southern mountainous region and the Eastern coastal area, connecting with the midwestern and southeastern high-risk regions to form a hot zone. The hot zone intensified over time and spread to the center and north. The risk in the Central and Northern regions changed from low to high after 2008. The risk of oral cancer in the northern and southwestern urban regions remained low over time.

The corresponding APC map for oral cancer in men (Fig. 2) indicates that the risk of oral cancer in Taiwan rose rapidly before 2005. The Central and Southern mountainous region and the Eastern coastal area had APC hot spots between 2001 and 2004, coinciding with the SIR hot zone formation mentioned above. After 2005, the APC in the whole of Taiwan began to decline, indicating that the increase in the risk of oral cancer had slowed down. The APC in the Central and Northern regions first decreased and then increased, indicating that the high-risk area of oral cancer had spread to the north. After 2015, the APC in Central and Southern Taiwan dropped below zero, indicating that oral cancer risk there had peaked and then declined.

The SIR map for breast cancer in women (Fig. 3) reveals that before 2005, the whole population of Taiwan was at low risk of breast cancer, but the cold spots were gradually shrinking. In 2005, an area of slightly elevated risk first appeared in the north, and later, hot spots emerged one after another in the Western, northeastern, and southeastern regions. These hot spots intensified and spread outward over time. Note that the high-risk areas of breast cancer tend to be concentrated in densely populated urban regions in the west. In contrast, the risks in the Western and Eastern coastal areas and the Central mountainous region remain low.

The APC map for breast cancer in women (Fig. 4) indicates that the risk of breast cancer in Taiwan has been rising over the last 21 years. The APC hot spots in the Northern region between 2005 and 2008 corresponded to the emergence and growth of the SIR hot spots in that region. The APC varied greatly over space and time, with the APC hot spots mostly occurring in the Western and Eastern urban areas and the Southern mountainous region. After 2011, the APC stabilized, although an APC hot spot appeared briefly in the southeastern region.

The spatiotemporal dynamic maps (Additional file 1: Appendices 4 and 5) clarify our understanding of the nuances and trends of disease risk dynamics.

## Discussion

The stabilized spatiotemporal kriging proposed in this study breaks the constraints of geopolitical boundaries and time intervals and stabilizes the disease rates properly. Hsu et al. [22] proposed the stabilized kriging method for spatial mapping. Their simulation results showed the benefits of the stabilized kriging method for spatial mapping of disease rates over the empirical Bayes and the traditional kriging methods. The spatiotemporal mapping (proposed in this study) is similar to the stabilized kriging method by Hsu et al.; however, in our proposed method, the additional variations in the time domain and those arising from spacetime interactions must also be considered. The simulation results of this study showed that the stabilized spatiotemporal kriging method improves on the traditional spatiotemporal kriging method when the spacetime interaction exists.

Disease rates are unstable in areas with small populations and appear extremely high or low. Therefore, the LAA-based spatiotemporal map of the original disease rates exhibits large jumps in values, even in adjacent LAAs or consecutive years (Additional file 1: Appendices 6 and 7). The traditional spatiotemporal kriging without adjusting for the nugget effect (assuming no measurement errors) can produce a smooth curved surface of the cross-sectional disease rates at any time point. However, the kriging estimate for a spatiotemporal centroid point (the centroid of an LAA in the middle of a year) is the original rate of the LAA in that year without a stabilizing effect (Additional file 1 :Appendices 8 and 9). At the other extreme, the nugget-adjusted spatiotemporal kriging (with homoscedasticity assumption) stabilizes the rates indiscriminately; it oversmooths the rates in populous regions but undersmooths the rates in sparsely populated ones (Additional file 1: Appendices 10 and 11). The stabilized spatiotemporal kriging adjusts the smoothing of a data point according to its variance, resulting in a desirable effect (Figs. 1 and 3). In this study, the stabilized spatiotemporal kriging is anchored to the centroids. The method could be extended to an area-to-area or area-to-point kriging [26, 27] in future research to consider the shapes, sizes, and population distributions of LAAs. In addition, the proposed method assumed a constant effect ($\mu$) across space and time, which was only suitable for a simple spatiotemporal process scenario. Kriging methods with the spatiotemporal trends fitted by straight lines, polynomials, or splines are also worthy of study.

The SIR map for oral cancer in men (Fig. 1) created by our proposed method reveals that the high-risk areas of oral cancer originated in the midwestern and southeastern regions and spread to the middle and the north, with persistent cold spots in the northern and southwestern urban regions. The spatiotemporal variations of oral cancer risk may be related to the Taiwanese habit of chewing betel nuts and concentrations of heavy metals in the soils [22, 28]. However, the SIR map for breast cancer in women (Fig. 3) reveals that the high-risk areas for breast cancer are concentrated in densely populated urban regions in the west, where people primarily have higher socioeconomic status and have adopted westernized lifestyles and eating habits [29]. Further studies can extend the methodology to the setting of the universal kriging (regression kriging) [9] to incorporate covariate information, if available, into spatiotemporal maps.

The stabilized spatiotemporal kriging has greater uncertainty in areas with smaller populations. To demonstrate the effect, we used the SIR hot spot for breast cancer in women in the mountainous, sparsely populated southeast region as an example (Fig. 3). We produced a SIR time-series plot for the hot spot centroid (Yanping Township in Taitung County). We did the same for a hot spot centroid in the densely populated Taipei Metropolis (Zhongzheng District in Taipei City). In Additional file 1: Appendix 12, the comparison reveals that the SIR trends are similar in the two LAAs, but the 95% confidence interval for the Yanping Township is much wider than that in the Zhongzheng District. Including 1.00 in the 95% SIR confidence interval for the Yanping Township raises whether it is genuinely a SIR hot spot.

Spatiotemporal maps facilitate the understanding of disease risk dynamics. We recommend using the stabilized spatiotemporal kriging method for mapping disease rates across space and time.

## Supplementary Information

**Additional file 1: Appendix 1.** Derivation of the variance of measurement error. **Appendix 2.** Taiwan island used in the simulation. **Appendix 3.** Simulation scenarios. **Appendix 4.** The spatiotemporal dynamic maps for the standardized incidence ratios (SIRs) and the annual percent changes (APCs) in the SIRs of oral cancer in men in Taiwan, 1997 to 2017. **Appendix 5.** The spatiotemporal dynamic maps for the standardized incidence ratios (SIRs) and the annual percent changes (APCs) in the SIRs of breast cancer in women in Taiwan, 1997 to 2017. **Appendix 6.** The local-administrative-area-based spatiotemporal map of the original disease rates of oral cancer in men in Taiwan, 1997 to 2017. **Appendix 7.** The local-administrative-area-based spatiotemporal map of the original disease rates of breast cancer in women in Taiwan, 1997 to 2017. **Appendix 8.** The traditional nugget-unadjusted spatiotemporal kriging map for the standardized incidence ratios (SIRs) of oral cancer in men in Taiwan, 1997 to 2017. **Appendix 9.** The traditional nugget-unadjusted spatiotemporal kriging map for the standardized incidence ratios (SIRs) of breast cancer in women in Taiwan, 1997 to 2017. **Appendix 10.** The traditional nugget-adjusted spatiotemporal kriging map for the standardized incidence ratios (SIRs) of oral cancer in men in Taiwan, 1997 to 2017. **Appendix 11.** The traditional nugget-adjusted spatiotemporal kriging map for the standardized incidence ratios (SIRs) of breast cancer in women in Taiwan, 1997 to 2017. **Appendix 12.** The time-series plot of the standardized incidence ratios (SIRs) of breast cancer in women in Taiwan, 1997 to 2017, for two hot spot centroids (Left: Zhongzheng District in Taipei City; Right: Yanping Township in Taitung County).

## Data Availability

Data are available from the National Taiwan Cancer Registry Database published by the Health Promotion Administration, Ministry of Health and Welfare of Taiwan. Due to legal restrictions imposed by the government of Taiwan in relation to the “Personal Information Protection Act”, data cannot be made publicly available. Requests for data can be sent as a formal proposal to the Health Promotion Administration, Ministry of Health and Welfare of Taiwan.
