# Supplementary material for: A stabilized spatiotemporal kriging method for disease mapping and application to male oral cancer and female breast cancer in Taiwan
Source: BMC Med Res Methodol. 2022 Oct 13;22:270. doi: 10.1186/s12874-022-01749-9 (PMC9563856; doi:10.1186/s12874-022-01749-9)

Appendix 1. Derivation of the variance of measurement error.

Assume $O_{ij} \sim Poisson(\lambda_{ij} = r_{ij} \times E_{ij})$ where O_{ij}, r_{ij}, and E_{ij} are the observed number of cases, the true value of the standardized incidence ratio, and the expected number of cases, respectively, in the ith LAA of the jth year. The maximum likelihood estimation of r_{ij} is $\hat{r}_{ij} = \frac{O_{ij}}{E_{ij}}$ and follows an asymptotic normal distribution $\hat{r}_{ij} \sim N\left(r_{ij}, \frac{r_{ij}}{E_{ij}}\right)$. By the delta method, $log(\hat{r}_{ij}) \sim N\left(log(r_{ij}), \frac{1}{\lambda_{ij}}\right)$. Then, the estimation of the variance of measurement error (v_{ij}) is $\hat{v}_{ij} = \frac{1}{\hat{\lambda}_{ij}} = \frac{1}{\hat{r}_{ij} \times E_{ij}} = \frac{1}{O_{ij}}$.

Appendix 2. Taiwan island used in the simulation.

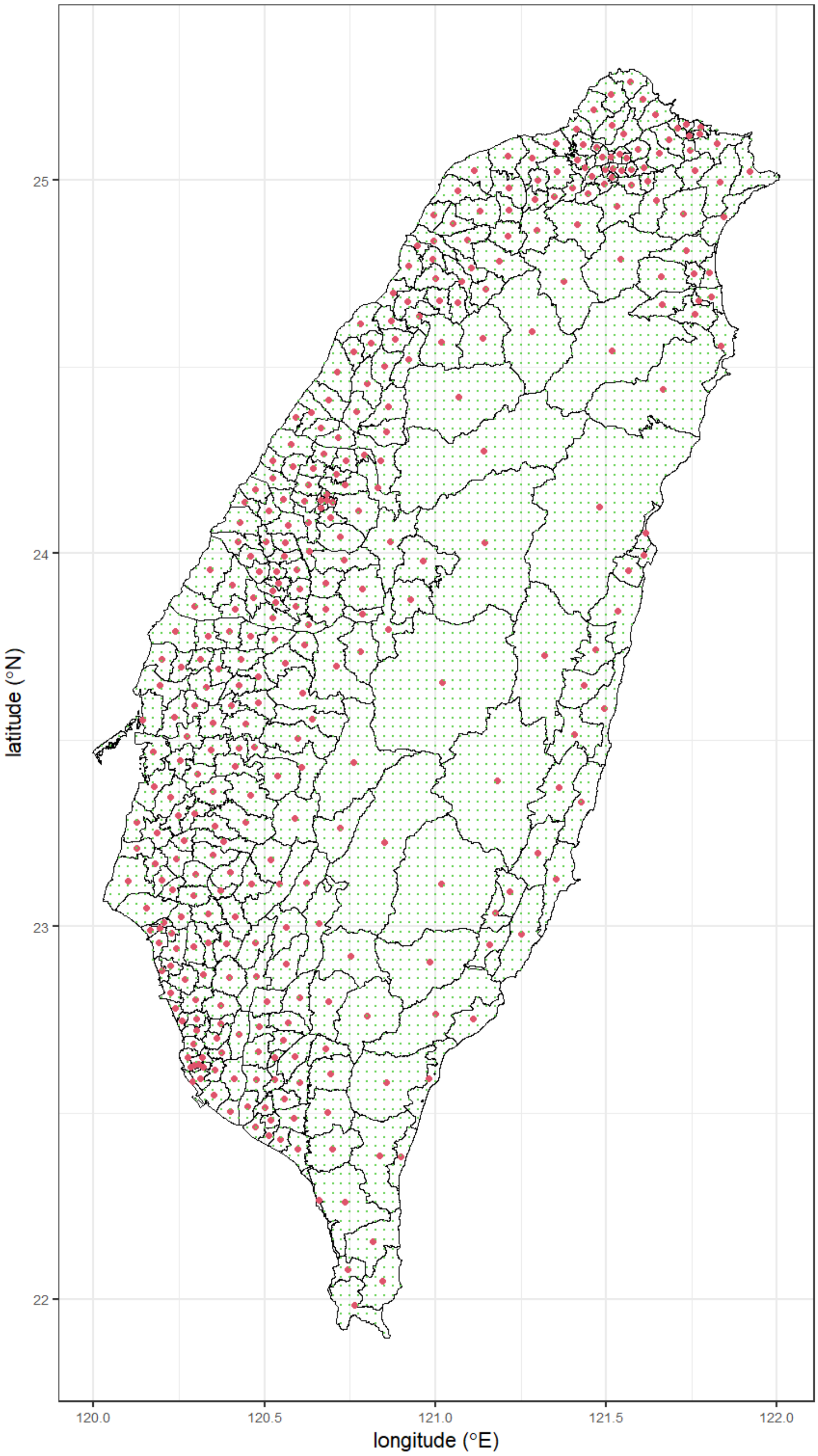

Appendix 3. Simulation scenarios.

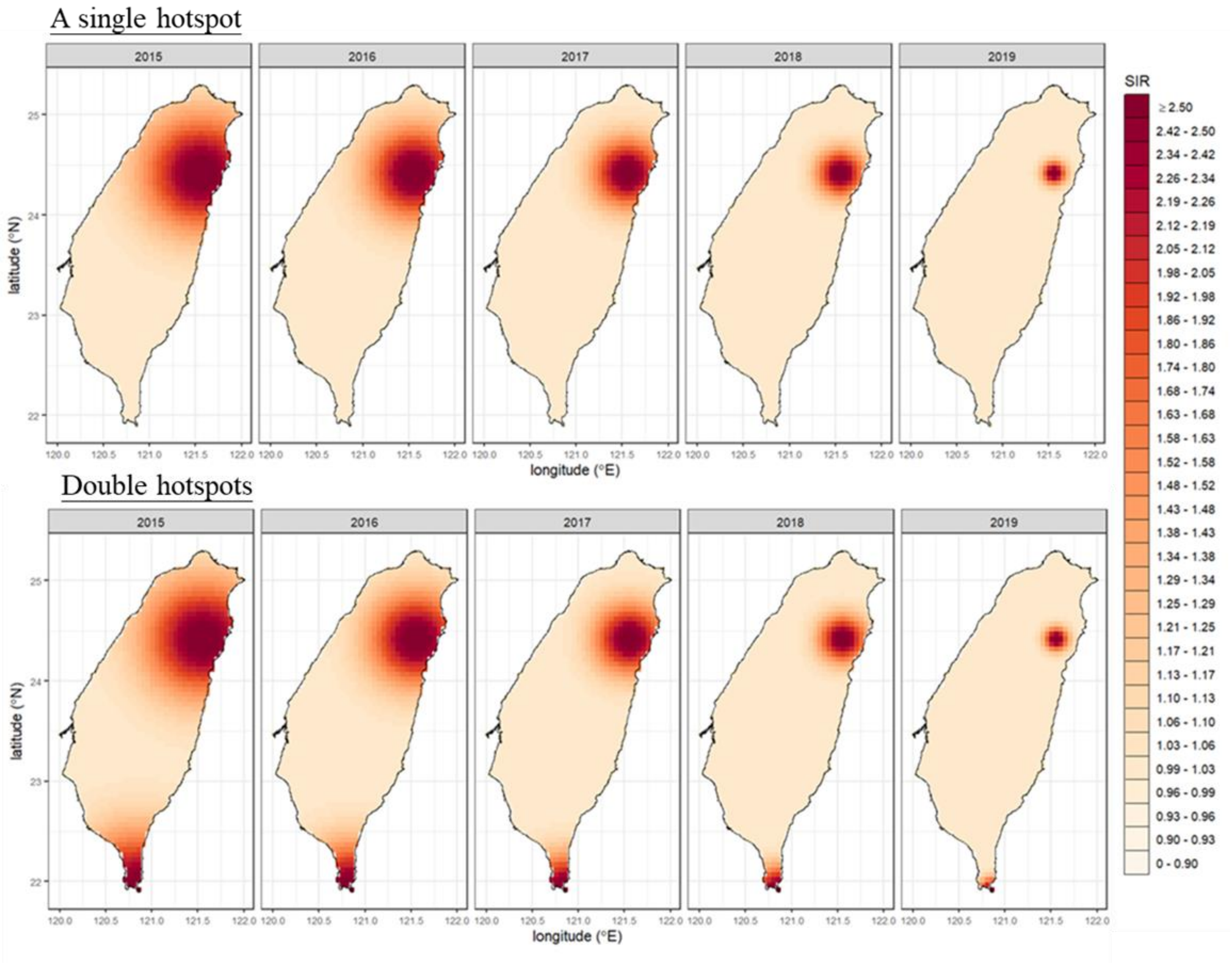

Appendix 4. The spatiotemporal dynamic maps for the standardized incidence ratios (SIRs) and the annual percent changes (APCs) in the SIRs of oral cancer in men in Taiwan, 1997 to 2017.

https://drive.google.com/file/d/18YNYaILS0xtCt4JI8FWWHR_Dm3GNhE4B/view?usp=sharing

Appendix 5. The spatiotemporal dynamic maps for the standardized incidence ratios (SIRs) and the annual percent changes (APCs) in the SIRs of breast cancer in women in Taiwan, 1997 to 2017.

https://drive.google.com/file/d/10qNDEkC9hA9L8o3vfuTIdBTQ86xWSrHK/view?usp=sharing

Appendix 6. The local-administrative-area-based spatiotemporal map of the original disease rates of oral cancer in men in Taiwan, 1997 to 2017.

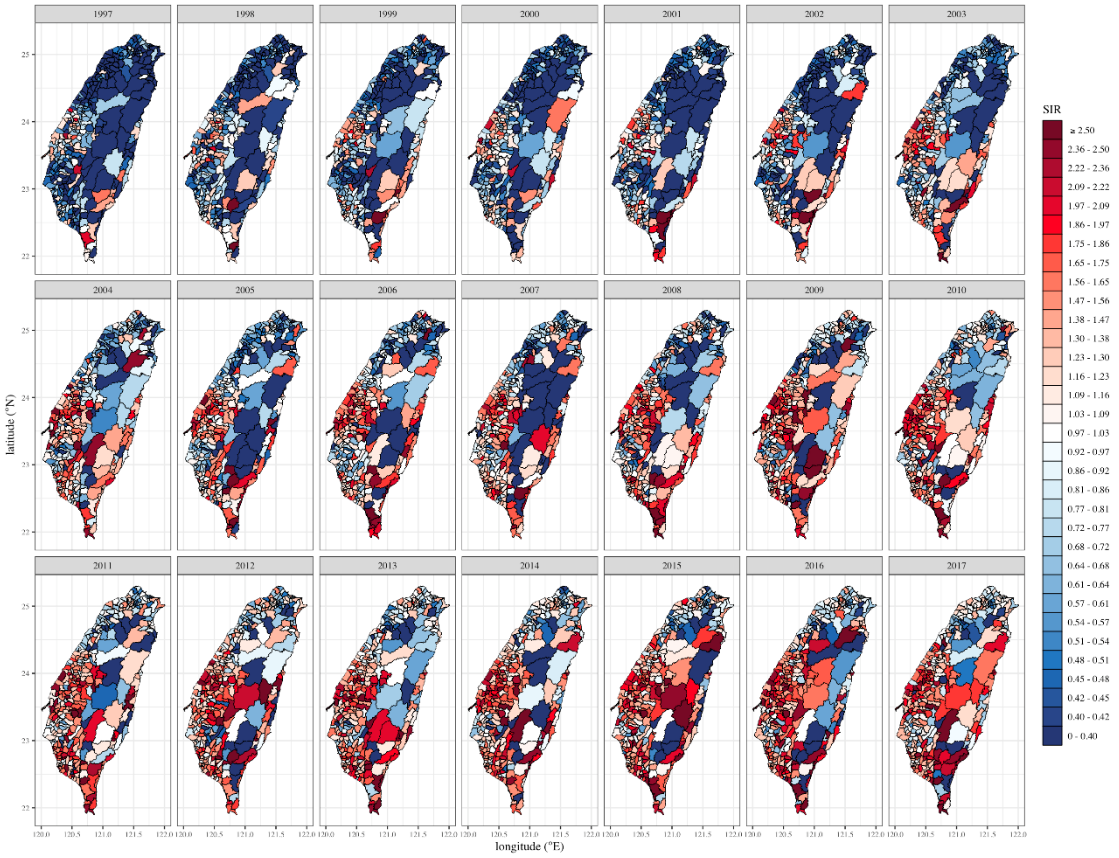

Appendix 7. The local-administrative-area-based spatiotemporal map of the original disease rates of breast cancer in women in Taiwan, 1997 to 2017.

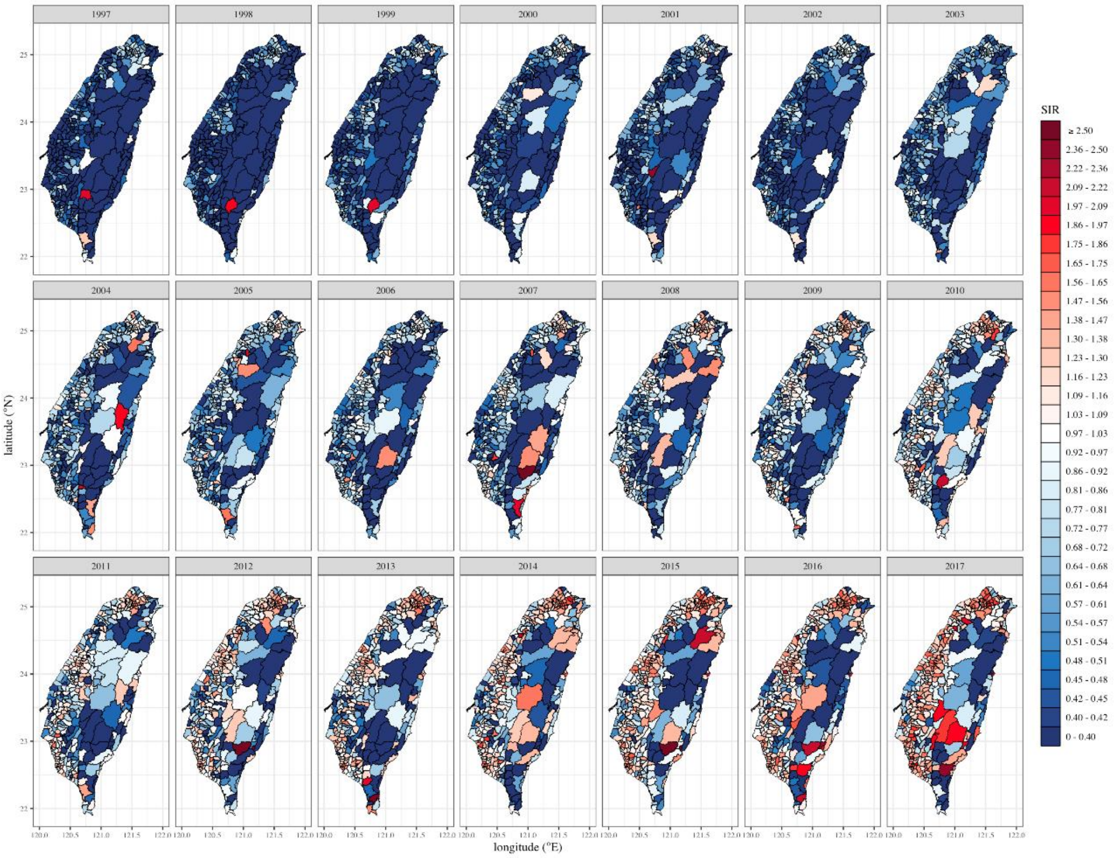

Appendix 8. The traditional nugget-unadjusted spatiotemporal kriging map for the standardized incidence ratios (SIRs) of oral cancer in men in Taiwan, 1997 to 2017.

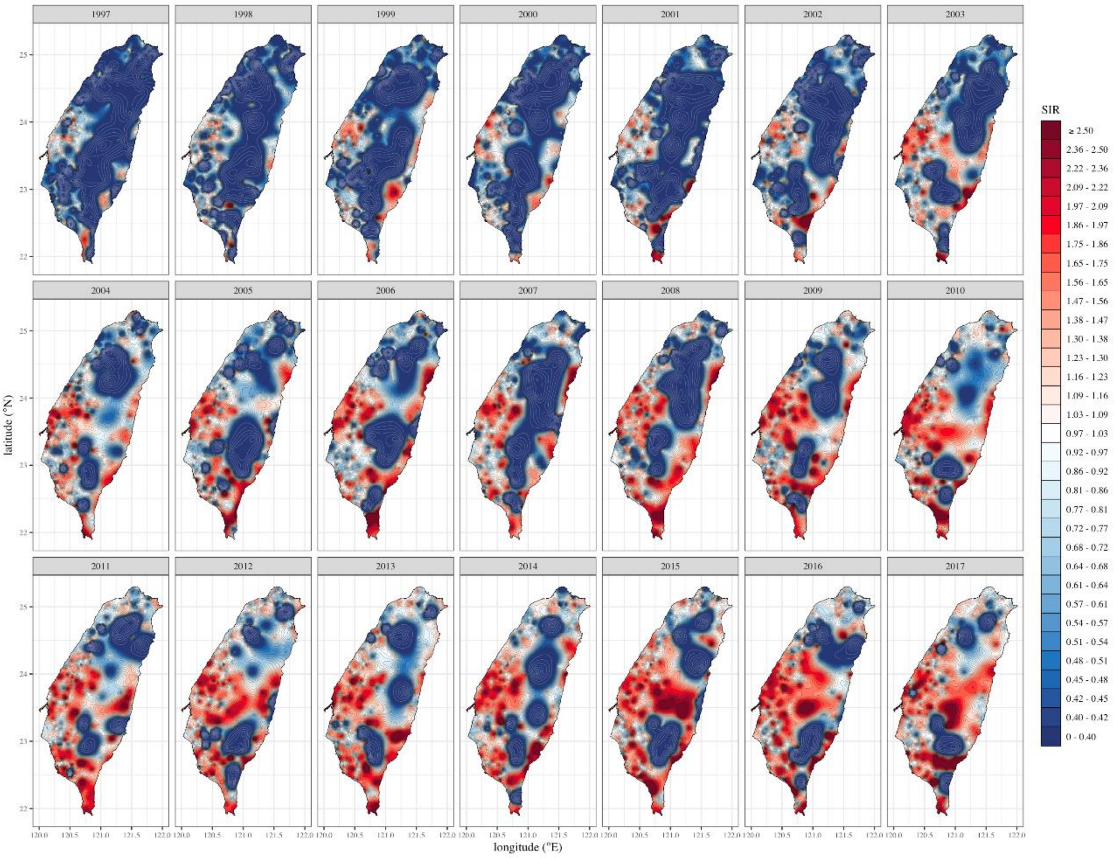

Appendix 9. The traditional nugget-unadjusted spatiotemporal kriging map for the standardized incidence ratios (SIRs) of breast cancer in women in Taiwan, 1997 to 2017.

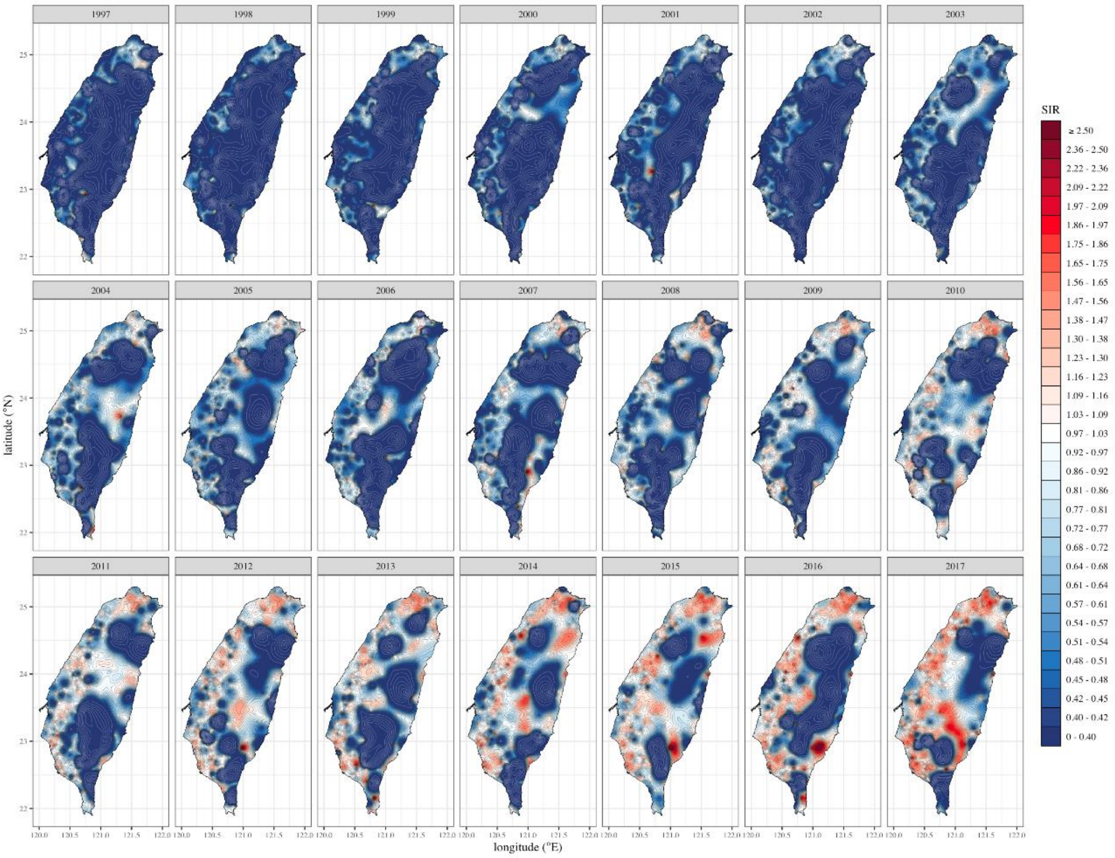

Appendix 10. The traditional nugget-adjusted spatiotemporal kriging map for the standardized incidence ratios (SIRs) of oral cancer in men in Taiwan, 1997 to 2017.

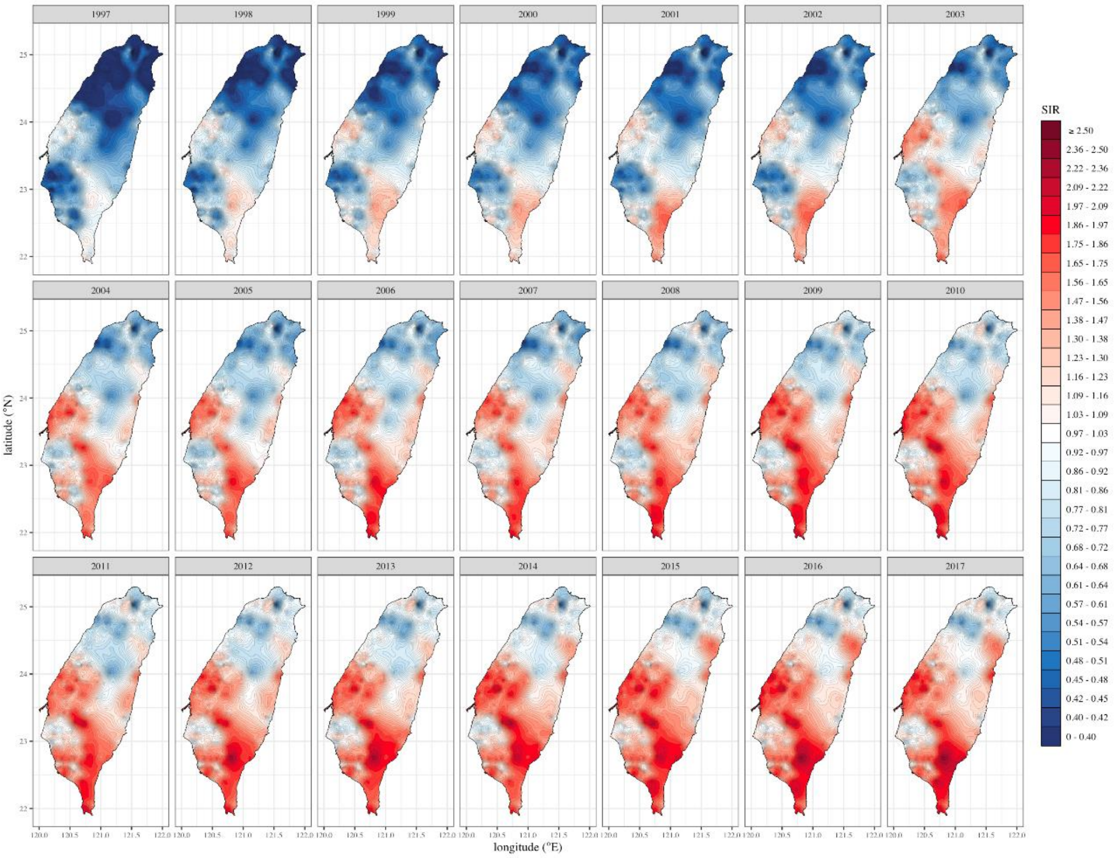

Appendix 11. The traditional nugget-adjusted spatiotemporal kriging map for the standardized incidence ratios (SIRs) of breast cancer in women in Taiwan, 1997 to 2017.

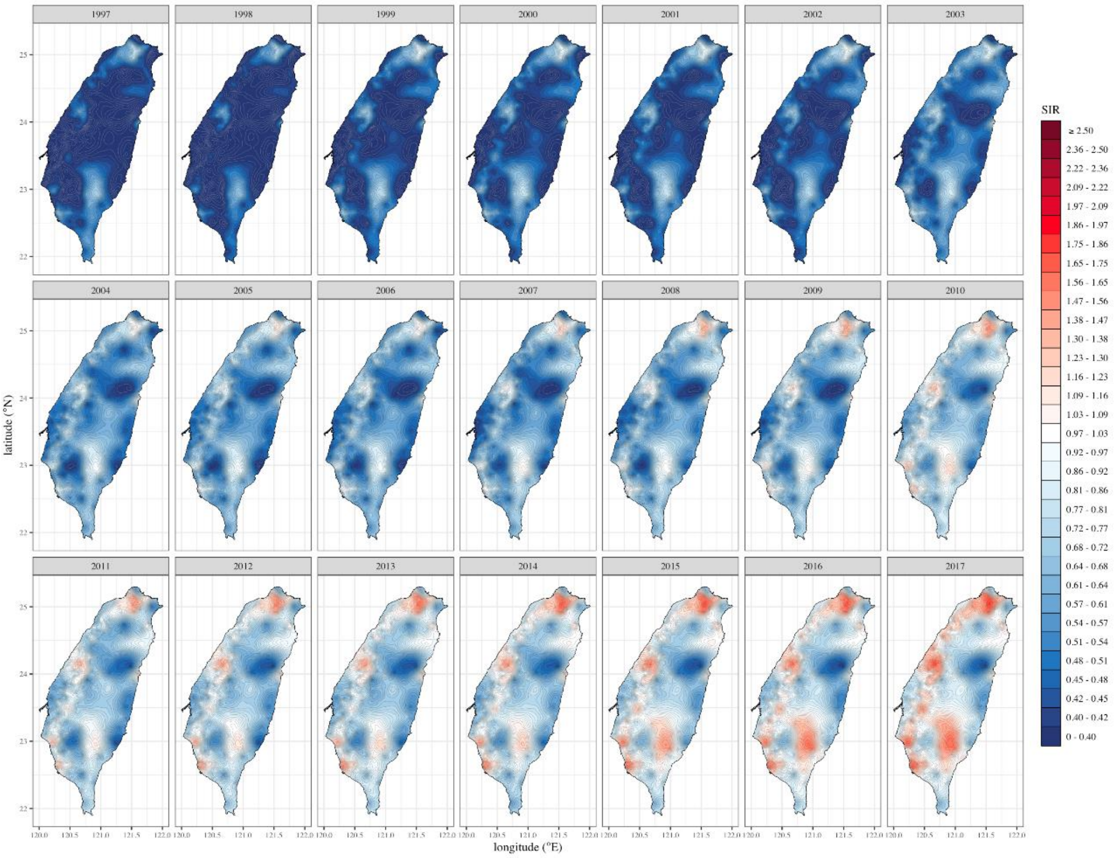

Appendix 12. The time-series plot of the standardized incidence ratios (SIRs) of breast cancer in women in Taiwan, 1997 to 2017, for two hot spot centroids (Left: Zhongzheng District in Taipei City; Right: Yanping Township in Taitung County).

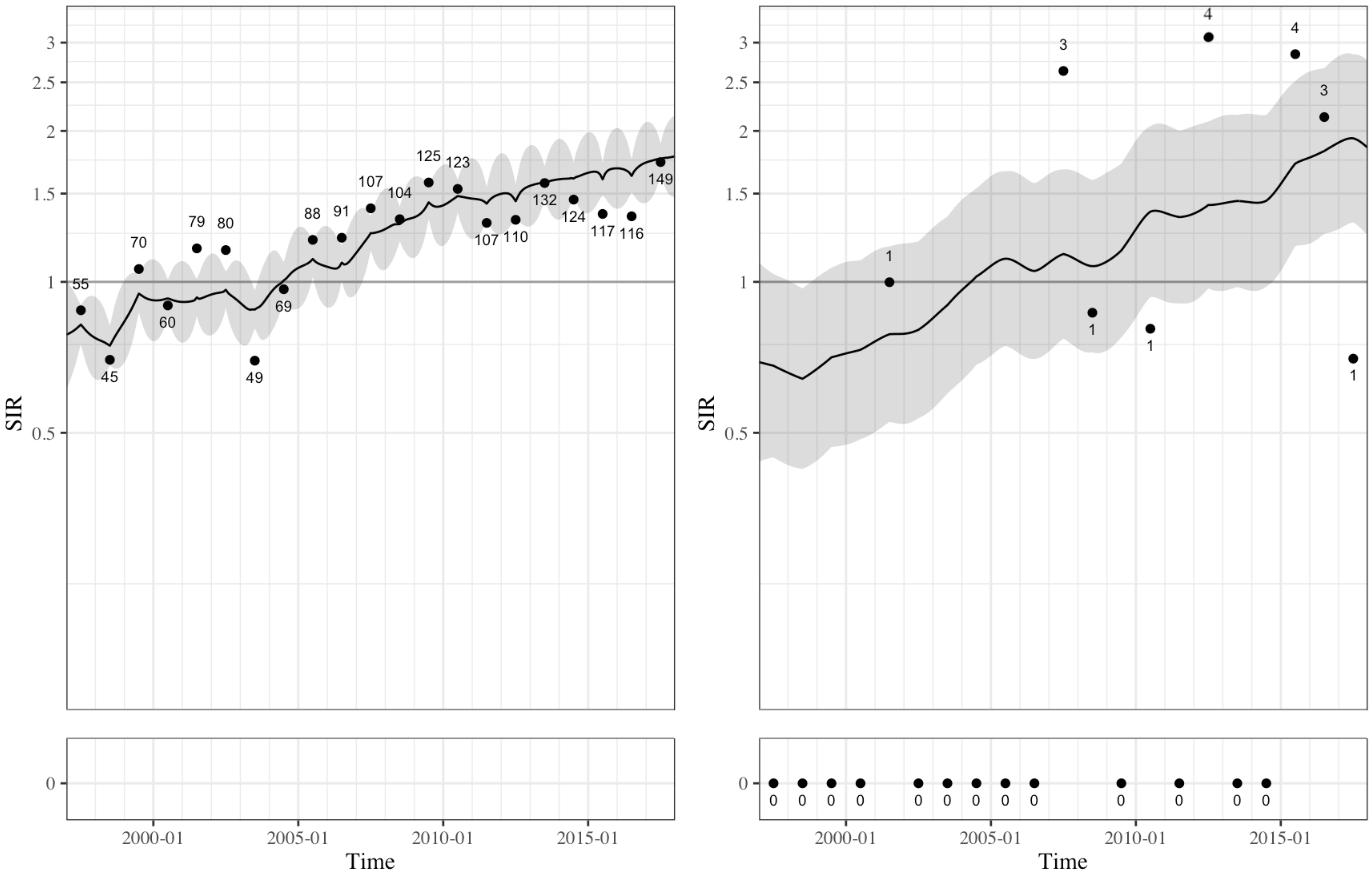

Supplement: Supplementary file 1 — Additional file 1: Appendix 1. Derivation of the variance of measurement error. Appendix 2. Taiwan island used in the simulation. Appendix 3. Simulation scenarios. Appendix 4. The spatiotemporal dynamic maps for the standardized incidence ratios (SIRs) and the annual percent changes (APCs) in the SIRs of oral cancer in men in Taiwan, 1997 to 2017. Appendix 5. The spatiotemporal dynamic maps for the standardized incidence ratios (SIRs) and the annual percent changes (APCs) in the SIRs of breast cancer in women in Taiwan, 1997 to 2017. Appendix 6. The local-administrative-area-based spatiotemporal map of the original disease rates of oral cancer in men in Taiwan, 1997 to 2017. Appendix 7. The local-administrative-area-based spatiotemporal map of the original disease rates of breast cancer in women in Taiwan, 1997 to 2017. Appendix 8. The traditional nugget-unadjusted spatiotemporal kriging map for the standardized incidence ratios (SIRs) of oral cancer in men in Taiwan, 1997 to 2017. Appendix 9. The traditional nugget-unadjusted spatiotemporal kriging map for the standardized incidence ratios (SIRs) of breast cancer in women in Taiwan, 1997 to 2017. Appendix 10. The traditional nugget-adjusted spatiotemporal kriging map for the standardized incidence ratios (SIRs) of oral cancer in men in Taiwan, 1997 to 2017. Appendix 11. The traditional nugget-adjusted spatiotemporal kriging map for the standardized incidence ratios (SIRs) of breast cancer in women in Taiwan, 1997 to 2017. Appendix 12. The time-series plot of the standardized incidence ratios (SIRs) of breast cancer in women in Taiwan, 1997 to 2017, for two hot spot centroids (Left: Zhongzheng District in Taipei City; Right: Yanping Township in Taitung County). [file 12874_2022_1749_MOESM1_ESM.pdf]
